# Association between season and pregnancy outcomes in fresh embryo transfer cycles: a systematic review and meta-analysis

**DOI:** 10.3389/fpubh.2025.1660982

**Published:** 2025-09-11

**Authors:** Qiang Deng, Fengying Wu, Jun Wang, Jiamei Huang, Lu Yin, Zhaoyang Ye, Ling Li, Siqi Deng, Yanyan Zhu, Zhichun Jin

**Affiliations:** 1 Hubei University of Chinese Medicine, Wuhan, China; 2 Maternal and Child Health Hospital of Hubei Province Tongji Medical College, Huazhong University of Science and Technology, Wuhan, China; 3 Shangqiu Municipal Hospital, Shangqiu, China

**Keywords:** seasons, fresh embryo transfer cycles, fertilization *in vitro*, embryo transfer, meta-analysis

## Abstract

**Background:**

Infertility represents a major global public health challenge. Identifying modifiable factors to optimize the outcomes of assisted reproductive technology (ART) is therefore crucial. Seasonal exposure has been reported to be correlated with natural conception rates, birth patterns, and adverse pregnancy outcomes. However, the potential associations between seasonal exposure and pregnancy outcomes among women undergoing fresh embryo transfer cycles remain controversial. This study aims to determine whether an association exists between seasonal exposure and pregnancy outcomes following fresh embryo transfer cycles, thereby providing evidence-based guidance on whether seasonal considerations should be integrated into ART treatment planning.

**Methods:**

This review adhered to the PRISMA guidelines. Literature searches were conducted across seven major electronic databases. Data synthesis employed either fixed-effects models. Subgroup analyses were performed according to the Köppen climate classification. The risk of bias in the included studies was assessed using the Newcastle-Ottawa Scale (NOS). Associations are reported as odds ratios (ORs) with corresponding 95% confidence intervals (CIs), and heterogeneity was quantified using the I^2^ statistic.

**Results:**

A total of 19 retrospective studies encompassing 159,696 fresh embryo transfer cycles were included. Overall, no significant associations were found between season and clinical pregnancy or live birth. Subgroup analyses, however, revealed climate-specific variations: In Cwa (humid subtropical) climates, live birth rates were significantly higher in summer than in winter (OR = 1.05; 95% CI, 1.01–1.10; I^2^ = 0%, P heterogeneity < 0.05). In Dfb (warm–summer humid continental) climates, live birth rates were significantly higher in spring than in summer (OR = 1.07; 95% CI, 1.01–1.14).

**Conclusion:**

Seasonal variation exhibits no significant association with pregnancy outcomes following fresh embryo transfer cycles. The observed variations within specific climate subgroups may result from multifactorial influences and require further research for validation. Clinicians and patients can prioritize medical indications and personal readiness without concern that seasonal timing may adversely impact pregnancy outcomes.

**Systematic review registration:**

https://www.crd.york.ac.uk/PROSPERO/view/CRD420251077751, Identifier: CRD420251077751.

## 1 Introduction

Infertility represents a major global public health challenge, affecting approximately 15% of reproductive-aged couples (1). It imposes significant psychological and financial burdens on individuals and may contribute to long-term population structure imbalances. While assisted reproductive technology (ART) offers effective treatment options for couples seeking pregnancy, its overall success rate remains around 36%, constrained by multiple factors (2, 3). Consequently, identifying modifiable factors to optimize ART outcomes is crucial.

Climate change continues to escalate, exceeding critical thresholds and posing a global health threat with unprecedented potential risks to human reproduction (4). Research suggests that external environmental factors, particularly climate conditions exhibiting seasonal fluctuations, may exert potential influences on human reproductive function. These effects could occur through pathways such as impacting gamete quality, endocrine regulation, and endometrial receptivity (5–7). Seasonal exposure has been reported to correlate with natural conception rates, birth patterns, and adverse pregnancy outcomes (8–11). This evidence indicates that seasonality may represent a significant environmental variable affecting ART success.

Among various ART procedures, the fresh embryo transfer cycle provides a unique and ideal model for investigating associations between seasonal exposure and pregnancy outcomes. Within this cycle, key steps—from ovarian stimulation and oocyte retrieval to fertilization and embryo transfer—occur within a relatively concentrated time frame (12). Crucially, embryos are transferred directly into the uterus without undergoing cryopreservation. This means that the critical early embryonic development and implantation phases are directly exposed to prevailing seasonal environmental factors. This model minimizes confounding effects from cryopreservation/thawing processes or extended time intervals between treatment stages, enabling a more direct and reliable assessment of the impact of specific seasonal exposures on pregnancy establishment.

Existing research on the influence of seasonal exposure on pregnancy outcomes for women undergoing fresh embryo transfer cycles shows significant inconsistencies. Some studies suggest an association between season and outcomes; for instance, a UK study in Liverpool observed significantly higher pregnancy rates following transfers performed in summer (13). Similarly, two studies from China reported significantly higher clinical pregnancy rates in spring and summer compared to winter (14, 15). Conversely, other studies found no such association, with research from both China and Turkey reporting no significant variations in clinical pregnancy rates across different seasons (16, 17).

Given this uncertainty in the literature, we designed this systematic review and meta-analysis to clarify whether an association exists between seasonal exposure and pregnancy outcomes following fresh embryo transfer cycles. This work aims to determine whether specific seasons represent a limiting factor for improving embryo transfer success rates, thereby providing evidence-based guidance on whether seasonal considerations should be integrated into ART planning.

## 2 Methods

This systematic review protocol adheres to the Preferred Reporting Items for Systematic Reviews and Meta-Analyses (PRISMA 2020) guidelines (see Supplementary Table 1) (18). Furthermore, the study protocol has been prospectively registered with the International Prospective Register of Systematic Reviews (PROSPERO) under registration number CRD42025107751.

### 2.1 Search strategy

Two investigators (Q D and FY W) independently conducted the literature searches. Initial searches were performed in the electronic databases EMBASE, PubMed, Scopus, Cochrane Library, Wanfang, and CNKI using a combination of subject headings and free-text terms. The primary search keywords included “season,” “oocyte retrieval,” “*in vitro* fertilization,” and “embryo transfer.” The search timeframe spanned from each database’s inception through June 21, 2025. Additionally, we manually searched the reference lists of relevant original studies. The specific PUBMED search strategy is detailed in Supplementary Table 2. Any discrepancies encountered during the search process were resolved through consultation with a third researcher (ZC J) who was not involved in the initial procedures.

### 2.2 Inclusion and exclusion criteria

Two investigators (Q D and FY W) independently screened the studies. The inclusion criteria were defined according to the PECOS framework (population, exposure, comparison, outcome, study): (1) population: women undergoing fresh embryo transfer cycles, including *in vitro* fertilization (IVF) and intracytoplasmic sperm injection (ICSI); (2) exposure: spring, summer, autumn, or winter; (3) comparison: comparisons between different seasons (e.g., spring vs. summer, summer vs. autumn); (4) outcome: clinical pregnancy and live birth; (5) study: cohort studies and case–control studies. The exclusion criteria were as follows: (1) studies lacking appropriate comparison groups (e.g., due to non-standardized seasonal definitions); (2) duplicate publications (only the study with the longest follow-up or most complete data was included); (3) studies where full-text articles or essential data were inaccessible; and (4) conference abstracts.

### 2.3 Parameter definition

(1)  Season: A season was defined as a consecutive three-month period: spring, summer, autumn, or winter. To facilitate meta-analysis of studies from different geographical regions, seasons were standardized based on meteorological criteria, prioritizing Northern Hemisphere classifications. For studies conducted in the Southern Hemisphere, seasons were reclassified accordingly. Similarly, studies reporting data by calendar month were consolidated into seasonal groupings. On the basis of Northern Hemisphere meteorological definitions, the seasons in this study were categorized as follows: spring (March--May), summer (June--August), autumn (September--November), and winter (December--February).
(2)  Clinical pregnancy: Defined as the detection of an intrauterine gestational sac with fetal cardiac activity via transvaginal ultrasound 4–5 weeks after embryo transfer.
(3)  Live birth: Live birth was defined as the delivery of at least one live infant at ≥28 weeks of gestation.

### 2.4 Data extraction

Two investigators (Q D and FY W) independently reviewed all eligible studies and extracted the following data: (1) first author; (2) publication year; (3) geographic location and climate type of the study site; (4) study design; (5) study duration; (6) total number of embryo transfer cycles; (7) number of clinical pregnancies per season; and (8) number of live births per season. Article screening, quality assessment, and data extraction were performed via EndNote X9 software. The outcome data reported as percentages were converted to absolute numbers for extraction. For studies with missing data, the corresponding authors were contacted via email within a specified time frame to request (Supplementary information).

### 2.5 Risk of bias

The Newcastle–Ottawa Scale (NOS) was used to assess the methodological quality of the included studies. This validated tool evaluates studies across three domains: (1) selection of subjects (4 items; maximum of 4 stars); (2) comparability of study groups (1 item; maximum of 2 stars); and (3) ascertainment of outcome/exposure (3 items; maximum of 3 stars). Studies achieving ≤4 stars were classified as low quality, those with 5–6 stars as moderate quality, and studies scoring ≥7 stars (maximum 9) as high quality.

### 2.6 Statistical analysis

The primary climate type of each study center was classified according to the Köppen climate classification system (see Supplementary Table 3). Using climate type as a subgroup factor, we performed pooled effect size calculations and comparisons across the following six seasonal pairings: (1) Spring vs. Summer; (2) Spring vs. Autumn; (3) Spring vs. Winter; (4) Summer vs. Autumn; (5) Summer vs. Winter; and (6) Autumn vs. Winter. Heterogeneity was assessed using the Cochrane chi-square test and quantified with the I^2^ statistic; I^2^ values exceeding 50% indicated substantial heterogeneity. Both fixed-effects and random-effects models were employed for data synthesis. Associations are reported as odds ratios (ORs) with corresponding 95% confidence intervals (CIs). Statistical significance was defined as a *p* value < 0.05. Publication bias was evaluated via Egger’s test, where *p* > 0.05 suggested no significant bias. If significant publication bias is present, the trim and fill method will be employed to assess its impact on the conclusions. Sensitivity analyses utilized the leave-one-out method. All analyses were conducted via STATA software, version 18.0.

## 3 Results

### 3.1 Characteristics of the studies

**Figure 1:**
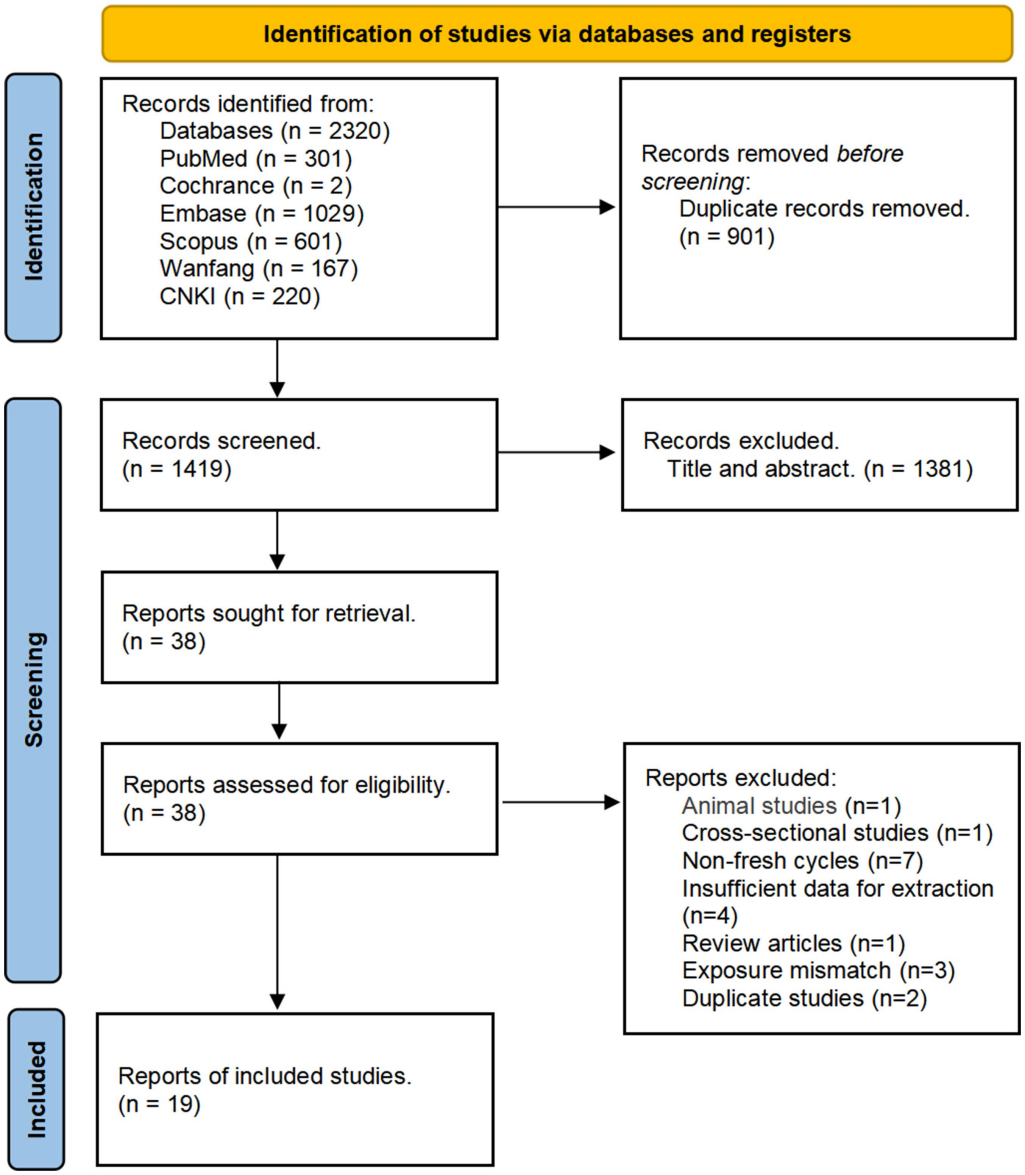
PRISMA flowchart of study selection.

Figure 1 shows the PRISMA flow diagram. Initial searches identified 2,320 records. After deduplication, 1,419 titles/abstracts were screened, yielding 38 full-text articles for eligibility assessment. Nineteen studies were excluded: animal studies (n = 1), cross-sectional studies (n = 1), non-fresh cycles (n = 7), insufficient data for extraction (n = 4), review articles (n = 1), exposure mismatches (n = 3), and duplicate studies (n = 2). A total of 19 studies were included in the quantitative analysis. Of these, 14 were published in English (13–15, 17, 19–29), and 4 were published in Chinese (30–33), spanning publication years from 1994 through 2025. The studies were primarily conducted in the following countries: Brazil, China, Iran, Israel, Italy, the Netherlands, Saudi Arabia, Sweden, Switzerland, Turkey, the United Kingdom, and the United States. The number of fresh embryo transfer cycles per study ranged from 266 to 52,788, with enrollment periods occurring between 1987 and 2023. Descriptive characteristics of the included studies are summarized in Table 1.

**Table 1:** Baseline characteristics of the studies included in the meta-analysis.

Author/Year	Study design	Location	Hemisphere	Köppen climate	Granularity	Fresh cycles	Total	Period	NOS score
Alzahrani et al. (2024) (28)	Retrospective Study	Aseer Region, Saudi Arabia	Northern	Bsh	Season	ICSI	2,194	2017.01–2021.09	8
Liu et al. (2019) (25)	Retrospective Study	Xian, China	Northern	Cwa	Season	IVF/ICSI	25,097	2014–2017	8
Chu et al. (2022) (14)	Retrospective Study	Zhengzhou, China	Northern	Cwa	Season	IVF	2,290	2015.06–2019.06	8
Wang et al. (2025) (15)	Retrospective Study	Nanning, China	Northern	Cfa	Season	IVF	1,179	2021.06–2023.10	8
Kirshenbaum, et al. (2018) (24)	Retrospective Study	Ramat Gan, Israel	Northern	Csa	Month	IVF	4,420	2013.06–2016.12	7
Li et al. (2025) (29)	Retrospective Study	Zhengzhou, China	Northern	Cwa	Season	IVF/ICSI	24,420	2011.01–2021.12	8
Farland et al. (2020) (26)	Retrospective Study	Boston, United States	Northern	Dfa	Season	ICSI	6,669	2012.01–2017.12	8
Braga et al. (2012) (23)	Retrospective Study	São Paulo, Brazil	Southern	Cfa	Season	ICSI	1,932	2005.01–2009.12	8
Korkmazn et al. (2023) (17)	Retrospective Study	Istanbul, Turkey	Northern	Csa	Month	ICSI	3,227	2007–2019	8
Carlsson Humla et al. (2022) (27)	Retrospective Study	Sweden	Northern	Dfb	Season	IVF/ICSI	52,788	2009–2018	8
Wunder et al. (2005) (21)	Retrospective Study	Switzerland	Northern	Cfb	Season	IVF	7,368	1995–2003	8
Revelli et al. (2005) (20)	Retrospective Study	Reggio Emilia, Italy	Northern	Cfa	Season	IVF	2,067	1998–2003	8
Stolwijk et al. (1994) (19)	Retrospective Study	Nijmegen, Netherlands	Northern	Cfb	Month	IVF	1,126	1987.02–1993.02	8
Xiao et al. (2018) (16)	Retrospective Study	Shanghai, China	Northern	Cfa	Season	ICSI	4,504	2010.01–2016.12	8
Chao et al. (30) (2016)	Retrospective Study	Wenzhou, China	Northern	Cfa	Season	IVF/ICSI	1,222	2010.01–2014.12	8
Du et al. (2023) (33)	Retrospective Study	Zhengzhou, China	Northern	Cwa	Season	IVF	14,446	2015.08–2019.10	8
Liu et al. (2018) (25)	Retrospective Study	Shenzhen, China	Northern	Cfa	Season	IVF/ICSI	1,772	2011.01–2017.02	8
Wood et al. (2006) (13)	Retrospective Study	Liverpool, United Kingdom	Northern	Cfb	Season	IVF/ICSI	2,709	1997.12–2001.11	8
Khafri et al. (2008) (22)	Retrospective Study	Tehran, Iran	Northern	Bsk	Season	ICSI	266	2005.07–2007.03	8

ICSI, Intracytoplasmic sperm injection; IVF, In vitro fertilization; NOS, Newcastle–Ottawa Scale.

### 3.2 Risk of bias

The quality assessment of the included studies is shown in Supplementary Table 4. All studies were rated “low risk of bias.”

### 3.3 Associations between season and clinical pregnancy

Data from 18 studies (13–15, 17, 19–25, 27–33) were available to assess the association between seasonal exposure and clinical pregnancy. As shown in Figure 2, heterogeneity testing revealed no significant heterogeneity; therefore, a fixed-effects model was used for the meta-analysis. The pooled overall effect sizes were as follows: (1) Spring vs. Summer: OR = 0.99 (95% CI, 0.96–1.02; I^2^ = 0%, P-heterogeneity > 0.05); (2) Spring vs. Autumn: OR = 1.00 (95% CI, 0.98–1.03; I^2^ = 0%, P-heterogeneity > 0.05); (3) Spring vs. Winter: OR = 1.01 (95% CI, 0.98–1.04; I^2^ = 0%, P-heterogeneity > 0.05); (4) Summer vs. Autumn: OR = 1.01 (95% CI, 0.98–1.04; I^2^ = 0%, P-heterogeneity > 0.05); (5) Summer vs. Winter: OR = 1.02 (95% CI, 0.99–1.05; I^2^ = 0%, P-heterogeneity > 0.05); (6) Autumn vs. Winter: OR = 1.01 (95% CI, 0.98–1.03; I^2^ = 0%, P-heterogeneity > 0.05). In the overall analysis, while the clinical pregnancy rates were slightly higher in summer and slightly lower in winter than in the other seasons, these findings were not statistically significant. Subgroup analysis on the basis of climate type also revealed no statistically significant differences in clinical pregnancy rates between seasons. Overall, no significant association was found between seasonal exposure and clinical pregnancy following fresh embryo transfer cycles.

**FGURE 2:**
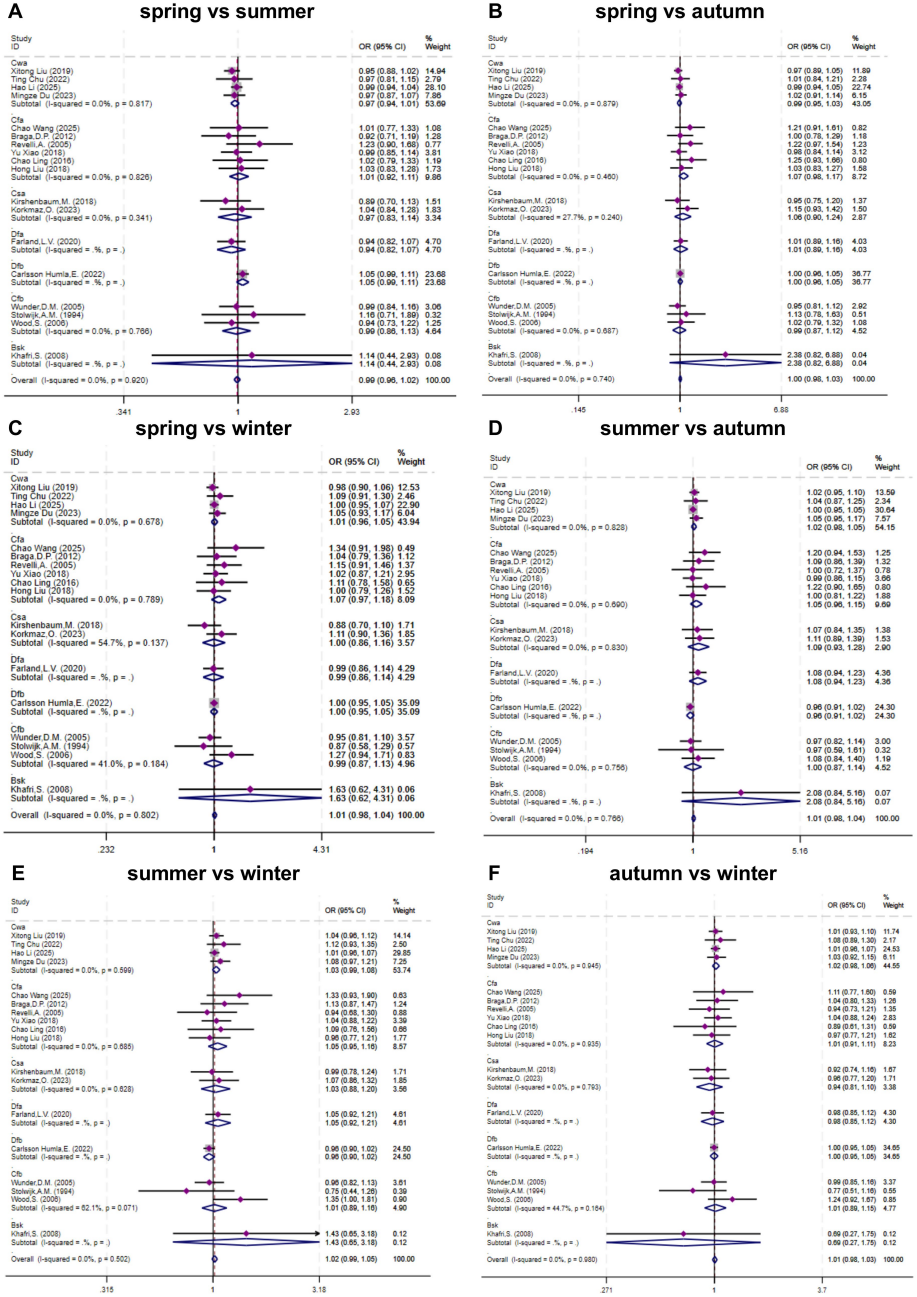
Meta-analysis forest plot of the associations between exposure to different seasons during fresh embryo transfer cycles and clinical pregnancy.

### 3.4 Association between season and live birth

**Figure 3:**
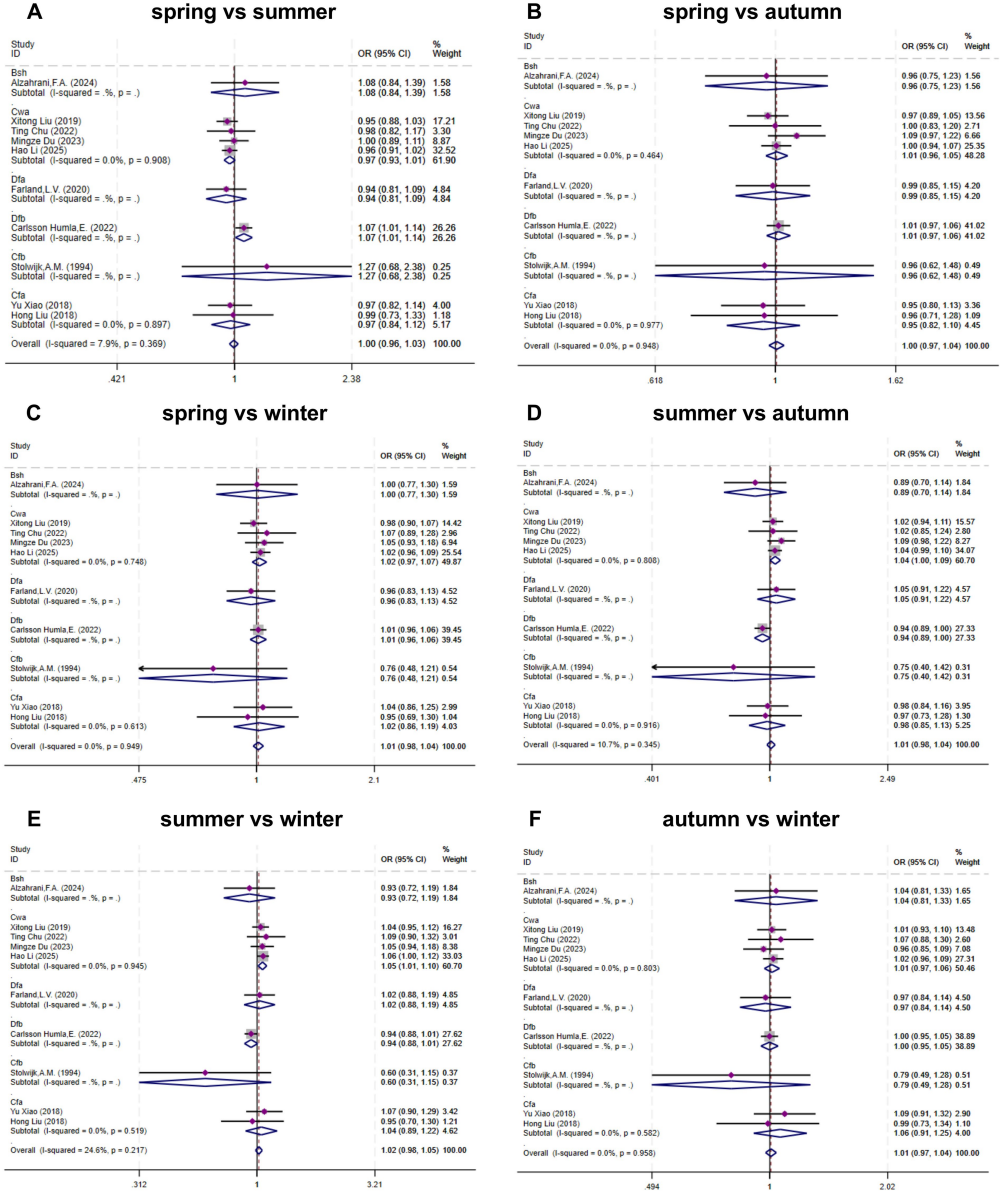
Meta-analysis forest plot of the associations between exposure to different seasons during fresh embryo transfer cycles and live birth.

Ten studies (14, 19, 25–29, 31–33) provided data to assess the association between seasonal exposure and live birth. As illustrated in Figure 3, heterogeneity testing revealed no significant heterogeneity; therefore, a fixed-effects model was employed for the meta-analysis. The pooled overall effect sizes were as follows: (1) Spring vs. Summer: OR = 1.00 (95% CI, 0.96–1.03; I^2^ = 7.9%, P-heterogeneity > 0.05); (2) Spring vs. Autumn: OR = 1.00 (95% CI, 0.97–1.04; I^2^ = 0%, P-heterogeneity > 0.05); (3) Spring vs. Winter: OR = 1.01 (95% CI, 0.98–1.04; I^2^ = 0%, P-heterogeneity > 0.05); (4) Summer vs. Autumn: OR = 1.01 (95% CI, 0.98–1.04; I^2^ = 10.7%, P-heterogeneity > 0.05); (5) Summer vs. Winter: OR = 1.02 (95% CI, 0.98–1.05; I^2^ = 24.6%, P-heterogeneity > 0.05); (6) Autumn vs. Winter: OR = 1.01 (95% CI, 0.97–1.04; I^2^ = 0%, P-heterogeneity > 0.05). In the overall analysis, live birth rates were slightly lower in winter compared to other seasons; however, no statistically significant differences were observed between seasons.

Subgroup analysis by climate type yielded the following significant findings: (1) In Cwa (humid subtropical) climates, live birth rates were significantly higher in summer compared to winter (OR = 1.05, 95% CI, 1.01–1.10; I^2^ = 0%, P-heterogeneity < 0.05). (2) In Dfb (warm-summer humid continental) climates, live birth rates were significantly higher in spring compared to summer (OR = 1.07, 95% CI, 1.01–1.14). No statistically significant differences in live birth rates between seasons were observed in other climate subgroups.

### 3.5 Publication bias and sensitivity analysis

Egger’s test indicated potential publication bias for three comparisons: spring vs. autumn (*p* < 0.05), spring vs. winter (p < 0.05), and summer vs. autumn (p < 0.05) in terms of clinical pregnancy outcomes. No significant bias was detected in the other seasonal pairings (see Supplementary Table 4; Figure 1). The trim-and-fill analysis across the three comparative groups revealed that despite the presence of significant publication bias, the corrected effect sizes exhibited minimal alterations, and the confidence intervals remained crossing the line of null effect (LogOR = 0). This indicates that publication bias is unlikely to overturn the original conclusions (see Supplementary Figure 2). Leave-one-out sensitivity analysis confirmed the robustness of all effect estimates, with no substantial changes observed upon sequential study exclusion (see Supplementary Figures 3, 4).

## 4 Discussion

This systematic review incorporated 19 observational studies from multiple centers worldwide, encompassing a total of 159,696 fresh embryo transfer cycles. Overall analysis revealed no significant association between seasonal exposure and pregnancy outcomes following fresh embryo transfer. Although subgroup analyses yielded divergent conclusions, these findings require cautious interpretation.

Previous hypotheses suggested that seasons with longer daylight hours (e.g., summer) might improve IVF/ICSI outcomes by elevating serum vitamin D levels (34, 35). However, the primary findings of this study did not confirm statistically significant seasonal differences: although clinical pregnancy and live birth rates were numerically slightly higher in summer, they did not reach statistical significance. A key explanation may lie in the routine use of vitamin D nutritional supplementation in assisted reproduction. Current evidence indicates that sufficient vitamin D levels (≥30 ng/mL) are associated with superior IVF outcomes, and meta-analyses demonstrate a linear positive correlation between vitamin D concentrations exceeding 60 nmol/L and pregnancy outcomes (36, 37). Furthermore, extensive studies confirm that vitamin D deficiency is linked to adverse pregnancy and perinatal risks (38–40). Against this backdrop, as vitamin D screening and supplementation increasingly become standard clinical practice, the impact of natural seasonal gradients in vitamin D levels is likely attenuated, reaching a plateau (41). Additionally, the widespread use of sun protection in modern lifestyles may further diminish the actual benefits of summer sun exposure, thereby neutralizing its potential advantages (42).

Melatonin represents another potential mediator of seasonal effects. Its secretion is regulated by light exposure, exhibiting distinct seasonal fluctuations with longer duration and higher peak levels in winter compared to summer. Research indicates that such seasonal variations in photoperiod not only influence GnRH neuronal activity by modulating neurotransmitter release but are also reflected in human pre-ovulatory follicular fluid and serum concentrations (43). Some studies suggest that photoperiod may regulate steroid hormone secretion through modulating melatonin changes, potentially subsequently affecting endometrial receptivity (29, 44). Meanwhile, meta-analyses have demonstrated that melatonin supplementation significantly improves clinical pregnancy rates in assisted reproductive technology (45). This implies that seasonal variations in melatonin could influence pregnancy outcomes by regulating sex hormone production and follicular quality, theoretically conferring an advantage in winter. However, the findings of the present study do not support this hypothesis. This discrepancy is primarily attributed to contemporary IVF protocols: the widespread use of GnRH analogs for pituitary desensitization, combined with high-dose exogenous gonadotropins (Gn) for ovarian stimulation, largely decouples follicular and oocyte development from seasonal variations. Furthermore, the extensive application of artificial cycle endometrial preparation protocols may further mitigate potential seasonal impacts on endometrial receptivity. Consequently, the effects of seasonal light exposure and its mediated melatonin fluctuations on fresh embryo transfer cycles are likely substantially attenuated.

Exposure to high temperatures has been confirmed to suppress spermatogenesis and negatively impact male fertility. Previous studies have observed seasonal fluctuations in semen parameters, characterized by significant declines in sperm concentration and the percentage of rapidly progressive motile sperm during summer and autumn, with recovery in winter. Semen quality is generally superior in spring and winter compared to summer (46, 47). Theoretically, such fluctuations could pose challenges to the outcomes of assisted reproductive technology (ART) procedures that rely on semen quality. However, the findings of this study also do not support this expectation. This is primarily attributable to stringent sperm optimization techniques that isolate and select the most motile sperm for fertilization. Particularly, intracytoplasmic sperm injection (ICSI) technology effectively safeguards fertilization success by enabling the precise selection and injection of a single high-quality spermatozoon through micromanipulation, even when the total count of motile sperm is seasonally limited. Thus, standardized sperm processing procedures and the application of ICSI technology significantly mitigate the potential impact of seasonal variations in semen parameters on ultimate ART outcomes.

It is noteworthy that although the primary analysis indicated no overall effect of seasonal factors on pregnancy outcomes, subgroup analyses revealed statistically significant yet clinically marginal differences within specific climate zones. In Cwa zones (studies from China), the live birth rate in summer was 5% higher than in winter, while in Dfb zones (a study from Sweden), the live birth rate in spring was 7% higher than in summer. These findings likely reflect underlying sociocultural factors rather than direct biological seasonal effects. For the Cwa climate zone, where all included studies were conducted in China, the winter season coincides with the Lunar New Year period. Previous studies have indicated that lifestyle changes during this holiday—such as unhealthy dietary habits, smoking, alcohol consumption, sleep disruption, and emotional stress—may negatively impact live birth rates in IVF/ICSI cycles, potentially explaining the relatively lower outcomes observed in winter (48–50). Furthermore, for the Dfb climate zone, the sole relevant study from Sweden reported a substantial reduction in IVF cycle numbers during summer due to clinic closures and limited resources during holiday periods. This selection bias may have underestimated the true summer effect, thereby amplifying the apparent advantage of spring (27). Therefore, these subgroup results should be interpreted with caution. They suggest that in certain regions, strong behavioral patterns or healthcare system characteristics may mask or confound potential biological seasonal effects.

Furthermore, the highly standardized procedures and continuous technological advancements in modern assisted reproductive laboratories provide a stable *in vitro* environment for embryonic development, effectively insulating it from seasonal external fluctuations. Although the studies included in this analysis span over three decades and represent different technological generations, the pooled analysis revealed no significant seasonal effects, indicating that any potential seasonal influences present in earlier periods have been effectively mitigated by contemporary techniques. This conclusion is further supported by the fact that rigorous practices such as temperature control, air quality management, and culture system optimization have been standard in ART laboratories for more than 30 years (51).

The evidence from this study indicates no significant association between seasonal factors and pregnancy outcomes following fresh embryo transfer cycles, and season itself is not an independent risk factor for embryo transfer. Under well-established embryo transfer protocols and stringent laboratory environmental controls, the potential impact of seasonal fluctuations on key procedures—such as gamete handling, embryo culture, and selection—can be effectively minimized. Therefore, seasonal timing need not be incorporated into clinical decision-making. Physicians and patients should prioritize medical indications, individualized physiological status, and psychosocial readiness when determining the optimal timing for embryo transfer. This conclusion may also help alleviate patient anxiety regarding seasonal scheduling and promote the rational allocation of reproductive medical resources.

This study possesses several strengths: adherence to PRISMA reporting guidelines and prospective PROSPERO registration ensured methodological rigor. The inclusion of 19 studies from diverse global centers, all rated as high quality, enhances the generalizability of the findings. Subgroup analyses based on specific climate types were conducted to explore climate-specific seasonal effects. To our knowledge, this is the first meta-analysis addressing this topic. However, several limitations should be considered: due to the nature of retrospective studies, specific technical parameters were often not documented, and variations in the timing of technological advancements across centers, coupled with overlapping study periods, made it challenging to perform subgroup analyses on the impact of technological progress on seasonal sensitivity. The simplified definition of season may obscure the influence of specific meteorological parameters (e.g., extreme temperatures, environmental pollution, UV index). Additionally, the results do not address frozen–thawed embryo transfer cycles, warranting further investigation in the future.

## 5 Conclusion

Seasonal variation demonstrates no significant association with pregnancy outcomes following fresh embryo transfer cycles. Observed variations within specific climate subgroups may arise from multifactorial influences and require further validation through future research. Clinicians and patients can prioritize medical indications and personal readiness without concern that seasonal timing may adversely affect pregnancy outcomes.

## Data Availability

The original contributions presented in the study are included in the article/Supplementary material, further inquiries can be directed to the corresponding author.
