# Supplementary material for: Association between season and pregnancy outcomes in fresh embryo transfer cycles: a systematic review and meta-analysis
Source: Front Public Health. 2025 Sep 11;13:1660982. doi: 10.3389/fpubh.2025.1660982 (PMC12460414; doi:10.3389/fpubh.2025.1660982)

**SUPPLEMENTARY MATERIALS**

**Table S1.** PRISMA Checklist

| **Section and Topic** | **Item #** | **Checklist item** | **Location where item is reported** |
| --- | --- | --- | --- |
| **TITLE** | | |  |
| Title | 1 | Identify the report as a systematic review. | 1 |
| **ABSTRACT** | | |  |
| Abstract | 2 | See the PRISMA 2020 for Abstracts checklist. (made as per the Journal guidelines) | 2 |
| **INTRODUCTION** | | |  |
| Rationale | 3 | Describe the rationale for the review in the context of existing knowledge. | 2 |
| Objectives | 4 | Provide an explicit statement of the objective(s) or question(s) the review addresses. | 2 |
| **METHODS** | | |  |
| Eligibility criteria | 5 | Specify the inclusion and exclusion criteria for the review and how studies were grouped for the syntheses. | 4 |
| Information sources | 6 | Specify all databases, registers, websites, organisations, reference lists and other sources searched or consulted to identify studies. Specify the date when each source was last searched or consulted. | 4 |
| Search strategy | 7 | Present the full search strategies for all databases, registers and websites, including any filters and limits used. | Table S2 |
| Selection process | 8 | Specify the methods used to decide whether a study met the inclusion criteria of the review, including how many reviewers screened each record and each report retrieved, whether they worked independently, and if applicable, details of automation tools used in the process. | 4 |
| Data collection process | 9 | Specify the methods used to collect data from reports, including how many reviewers collected data from each report, whether they worked independently, any processes for obtaining or confirming data from study investigators, and if applicable, details of automation tools used in the process. | 4，5 |
| Data items | 10a | List and define all outcomes for which data were sought. Specify whether all results that were compatible with each outcome domain in each study were sought (e.g., for all measures, time points, analyses), and if not, the methods used to decide which results to collect. | 4 |
|  | 10b | List and define all other variables for which data were sought (e.g., participant and intervention characteristics, funding sources). Describe any assumptions made about any missing or unclear information. | 4 |
| Study risk of bias assessment | 11 | Specify the methods used to assess risk of bias in the included studies, including details of the tool(s) used, how many reviewers assessed each study and whether they worked independently, and if applicable, details of automation tools used in the process. | 4，5 |
| Effect measures | 12 | Specify for each outcome the effect measure(s) (e.g. risk ratio, mean difference) used in the synthesis or presentation of results. | 8-11 |
| Synthesis methods | 13a | Describe the processes used to decide which studies were eligible for each synthesis (e.g. tabulating the study intervention characteristics and comparing against the planned groups for each synthesis (item #5)). | 5-7, Table 1 |
|  | 13b | Describe any methods required to prepare the data for presentation or synthesis, such as handling of missing summary statistics, or data conversions. | NA |
|  | 13c | Describe any methods used to tabulate or visually display results of individual studies and syntheses. | Table 1  Figure 1-3 |
|  | 13d | Describe any methods used to synthesize results and provide a rationale for the choice(s). If meta-analysis was performed, describe the model(s), method(s) to identify the presence and extent of statistical heterogeneity, and software package(s) used. | 5 |
|  | 13e | Describe any methods used to explore possible causes of heterogeneity among study results (e.g. subgroup analysis, meta-regression). | 5 |
|  | 13f | Describe any sensitivity analyses conducted to assess robustness of the synthesized results. | 5 |
| Reporting bias assessment | 14 | Describe any methods used to assess risk of bias due to missing results in a synthesis (arising from reporting biases). | 5 |
| Certainty assessment | 15 | Describe any methods used to assess certainty (or confidence) in the body of evidence for an outcome. | NA |
| **RESULTS** | | |  |
| Study selection | 16a | Describe the results of the search and selection process, from the number of records identified in the search to the number of studies included in the review, ideally using a flow diagram. | 5, Figure-1, |
|  | 16b | Cite studies that might appear to meet the inclusion criteria, but which were excluded, and explain why they were excluded. | 5, Figure-1  Table 1 |
| Study characteristics | 17 | Cite each included study and present its characteristics. | Table-1 |
| Risk of bias in studies | 18 | Present assessments of risk of bias for each included study. | 8, Table S4 |
| Results of individual studies | 19 | For all outcomes, present, for each study: (a) summary statistics for each group (where appropriate) and (b) an effect estimate and its precision (e.g. confidence/credible interval), ideally using structured tables or plots. | 5-11,  Figure1 -3 |
| Results of syntheses | 20a | For each synthesis, briefly summarise the characteristics and risk of bias among contributing studies. | 11 |
|  | 20b | Present results of all statistical syntheses conducted. If meta-analysis was done, present for each the summary estimate and its precision (e.g. confidence/credible interval) and measures of statistical heterogeneity. If comparing groups, describe the direction of the effect. | 8-11 |
|  | 20c | Present results of all investigations of possible causes of heterogeneity among study results. | NA |
|  | 20d | Present results of all sensitivity analyses conducted to assess the robustness of the synthesized results. | 11 |
| Reporting biases | 21 | Present assessments of risk of bias due to missing results (arising from reporting biases) for each synthesis assessed. | 11,Figure S2 |
| Certainty of evidence | 22 | Present assessments of certainty (or confidence) in the body of evidence for each outcome assessed. | NA |
| **DISCUSSION** | | |  |
| Discussion | 23a | Provide a general interpretation of the results in the context of other evidence. | 12-14 |
|  | 23b | Discuss any limitations of the evidence included in the review. | 12-14 |
|  | 23c | Discuss any limitations of the review processes used. | 12-14 |
|  | 23d | Discuss implications of the results for practice, policy, and future research. | 12-14 |
| **OTHER INFORMATION** | | |  |
| Registration and protocol | 24a | Provide registration information for the review, including register name and registration number, or state that the review was not registered. | 3 |
|  | 24b | Indicate where the review protocol can be accessed, or state that a protocol was not prepared. | 3 |
|  | 24c | Describe and explain any amendments to information provided at registration or in the protocol. | NA |
| Support | 25 | Describe sources of financial or non-financial support for the review, and the role of the funders or sponsors in the review. | 1 |
| Competing interests | 26 | Declare any competing interests of review authors. | 1 |
| Availability of data, code and other materials | 27 | Report which of the following are publicly available and where they can be found: template data collection forms; data extracted from included studies; data used for all analyses; analytic code; any other materials used in the review. | 1 |

**Table S2.** PUBMED (Searched on: June 21, 2025)

| Search number | Query | Results |
| --- | --- | --- |
| #1 | Seasons[MeSH Terms] | 128,129 |
| #2 | ((((Season[Title/Abstract]) OR (Seasonal Variation[Title/Abstract])) OR (Seasonal Variations[Title/Abstract])) OR (Variation, Seasonal[Title/Abstract])) OR (Variations, Seasonal[Title/Abstract]) | 121,730 |
| #3 | #1 OR #2 | 205085 |
| #4 | Oocyte Retrieval[MeSH Terms] | 2,599 |
| #5 | ((((Retrieval, Oocyte[Title/Abstract]) OR (Oocyte Collection[Title/Abstract])) OR (Collection, Oocyte[Title/Abstract])) OR (Oocyte Aspiration[Title/Abstract])) OR (Aspiration, Oocyte[Title/Abstract]) | 724 |
| #6 | Embryo Transfer[MeSH Terms] | 19,801 |
| #7 | ((((((((Embryo Transfers[Title/Abstract]) OR (Transfer, Embryo[Title/Abstract])) OR (Transfers, Embryo[Title/Abstract])) OR ([Title/Abstract])) OR (Tubal Embryo Stage Transfer[Title/Abstract])) OR (Blastocyst Transfer[Title/Abstract])) OR (In vitro fertilization embryo transfer[Title/Abstract])) OR (IVF-ET[Title/Abstract])) OR (IVF[Title/Abstract]) | 24,751 |
| #8 | Fertilization in Vitro[MeSH Terms] | 43,609 |
| #9 | ((((((((((((In Vitro Fertilization[Title/Abstract]) OR (In Vitro Fertilizations[Title/Abstract])) OR (Test-Tube Fertilization[Title/Abstract])) OR (Fertilizations, Test-Tube[Title/Abstract])) OR (Fertilization, Test-Tube[Title/Abstract])) OR (Test Tube Fertilization[Title/Abstract])) OR (Test-Tube Fertilizations[Title/Abstract])) OR (Fertilizations in Vitro[Title/Abstract])) OR (Test-Tube Babies[Title/Abstract])) OR (Babies, Test-Tube[Title/Abstract])) OR (Baby, Test-Tube[Title/Abstract])) OR (Test Tube Babies[Title/Abstract])) OR (Test-Tube Baby[Title/Abstract]) | 29,664 |
| #10 | #4 OR #5 OR #6 OR #7 OR #8 OR #9 | 62,838 |
| #11 | #3 AND #11 | 301 |

**Table S3.** Köppen climate classification.

| Climate Type Code | Specific Climate Type | Regions |
| --- | --- | --- |
| Bsh | Tropical semi-arid climate | Abha, SA |
| Bsk | Temperate semi-arid climate | Tehran, IR |
| Cfa | Humid subtropical climate (no dry season) | Nanning, CN、São Paulo, BR、Reggio Emilia, IT、Shanghai, CN、Wenzhou, CN、Shenzhen, CN |
| Cfb | Temperate oceanic climate | CH、Nijmegen, NL、Liverpool, UK |
| Csa | Mediterranean climate (hot, dry summer) | Ramat Gan, IL、Istanbul, TR |
| Cwa | Humid subtropical climate (dry winter) | Xi'an, CN、Zhengzhou, CN |
| Dfa | Humid continental climate (hot summer) | Boston, US |
| Dfb | Humid continental climate (warm summer) | SE |

**Table S4.** Quality assessment (Newcastle-Ottawa Scale [NOS]).

| Author | Year | Representativeness of the exposed cohort | Selection of non-exposed cohort | Ascertainment of exposure factor | Demonstration that outcome of interest was not present at start of study | Comparability of cohorts on the basis of the design or analysis (★★) | Evaluation of outcome | Was follow-up long enough for outcomes to occur | Adequacy of follow-up of cohorts | Quality Scores |
| --- | --- | --- | --- | --- | --- | --- | --- | --- | --- | --- |
| Alzahrani F.A. | 2024 | ★ | ★ | ★ | ★ | ★ | ★ | ★ | ★ | 8 |
| Xitong Liu | 2019 | ★ | ★ | ★ | ★ | ★ | ★ | ★ | ★ | 8 |
| Ting Chu | 2022 | ★ | ★ | ★ | ★ | ★ | ★ | ★ | ★ | 8 |
| Chao Wang | 2025 | ★ | ★ | ★ | ★ | ★ | ★ | ★ | ★ | 8 |
| Kirshenbaum, M. | 2018 | ★ | ★ | ★ | ★ | — | ★ | ★ | ★ | 7 |
| Hao Li | 2025 | ★ | ★ | ★ | ★ | ★ | ★ | ★ | ★ | 8 |
| Farland, L. V. | 2020 | ★ | ★ | ★ | ★ | ★ | ★ | ★ | ★ | 8 |
| Braga, D. P. | 2012 | ★ | ★ | ★ | ★ | ★ | ★ | ★ | ★ | 8 |
| Korkmaz, O. | 2023 | ★ | ★ | ★ | ★ | ★ | ★ | ★ | ★ | 8 |
| Carlsson Humla, E. | 2022 | ★ | ★ | ★ | ★ | ★ | ★ | ★ | ★ | 8 |
| Yu Xiao | 2018 | ★ | ★ | ★ | ★ | ★ | ★ | ★ | ★ | 8 |
| Wunder, D. M. | 2005 | ★ | ★ | ★ | ★ | ★ | ★ | ★ | ★ | 8 |
| Revelli, A. | 2005 | ★ | ★ | ★ | ★ | ★ | ★ | ★ | ★ | 8 |
| Stolwijk, A. M. | 1994 | ★ | ★ | ★ | ★ | ★ | ★ | ★ | ★ | 8 |
| Chao Lin | 2016 | ★ | ★ | ★ | ★ | ★ | ★ | ★ | ★ | 8 |
| Mingze Du | 2023 | ★ | ★ | ★ | ★ | ★ | ★ | ★ | ★ | 8 |
| Hong Liu | 2018 | ★ | ★ | ★ | ★ | ★ | ★ | ★ | ★ | 8 |
| Wood, S. | 2006 | ★ | ★ | ★ | ★ | ★ | ★ | ★ | ★ | 8 |
| Khafri, S. | 2008 | ★ | ★ | ★ | ★ | ★ | ★ | ★ | ★ | 8 |

Notes: “★” represents 1 point, “×” represents 0 point, and “—” represents uncertain points. Abbreviation: NOS, Newcastle–Ottawa Scale.

**Table S4.** Egger's test

| season | Clinical pregnancy (n=18) | | Live birth (n=10) | |
| --- | --- | --- | --- | --- |
|  | t | *p* | t | *p* |
| Spring vs Summer | 0.11 | 0.91 | -0.41 | 0.691 |
| Spring vs Autumn | 3.08 | 0.007 | -1.52 | 0.168 |
| Spring vs Winter | 2.27 | 0.038 | -1.64 | 0.139 |
| Summer vs Autumn | 3.46 | 0.003 | -0.93 | 0.382 |
| Summer vs Winter | 1.95 | 0.069 | -0.59 | 0.571 |
| Autumn vs Winter | -0.47 | 0.642 | 0.11 | 0.916 |

Note: P-value >0.05 indicated no publication bias

**Figure S1**.Clinical pregnancy-Egger's test


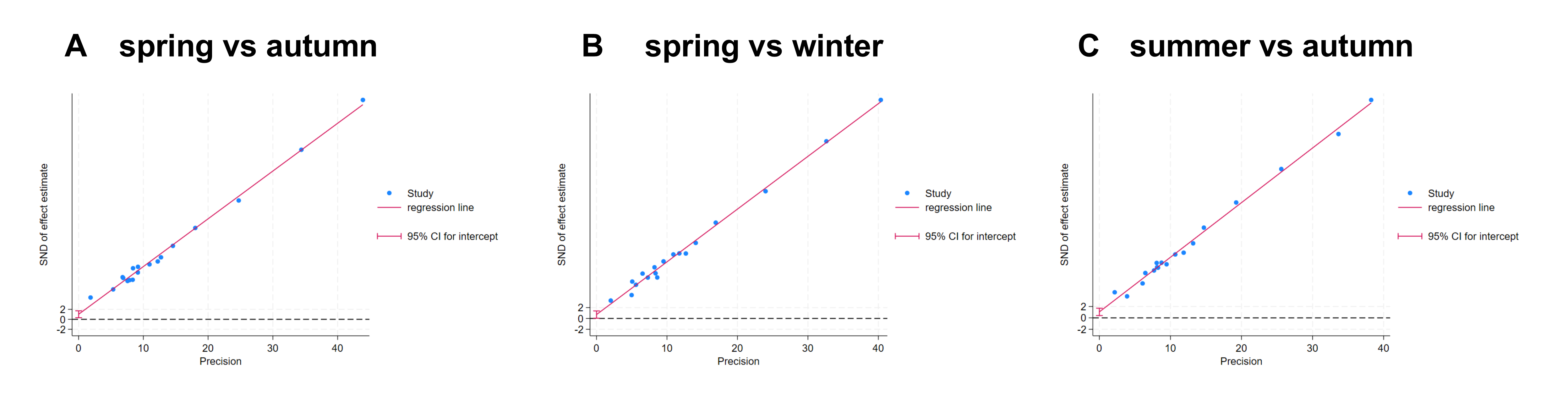


**Figure S2.** trim-and-fill analysis

1. spring vs. autumn


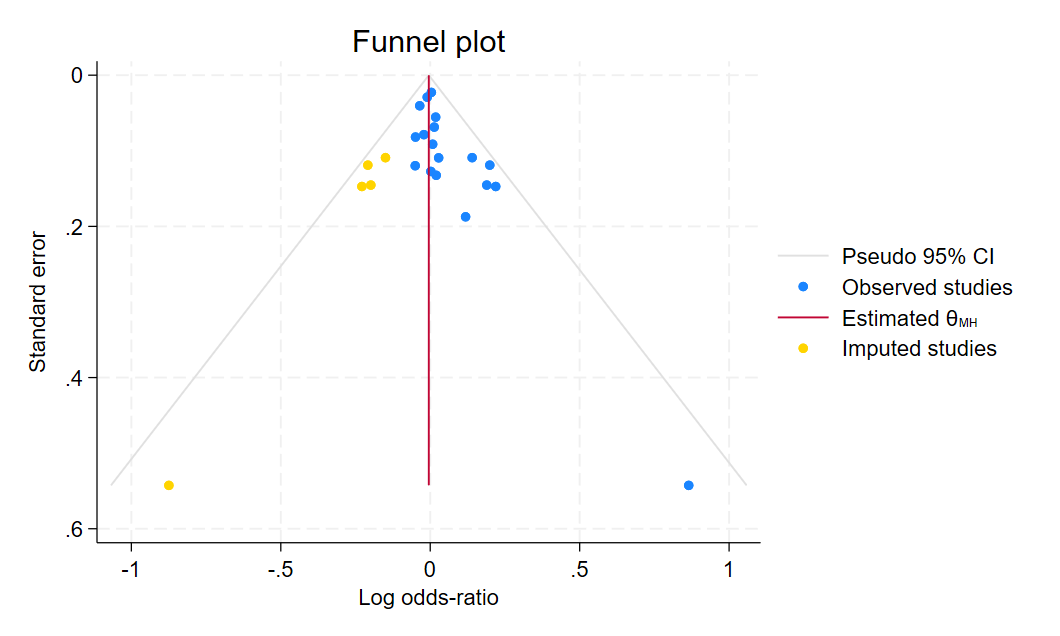


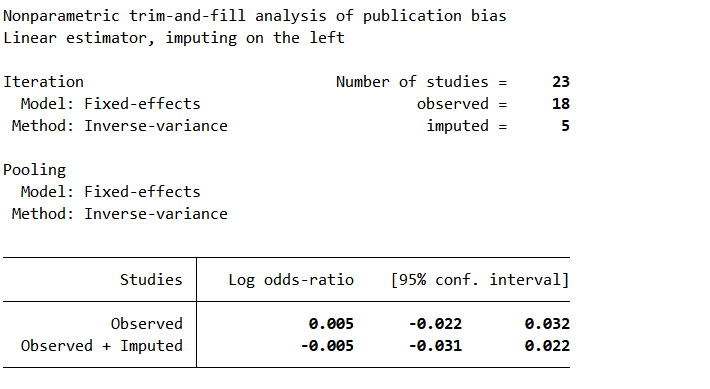


1. spring vs. winter

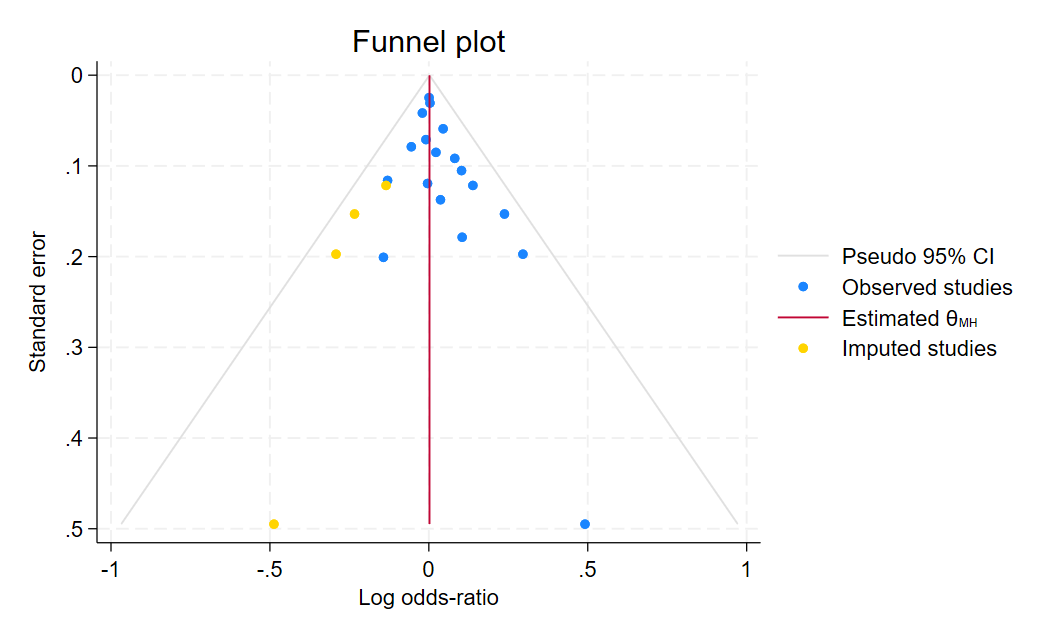


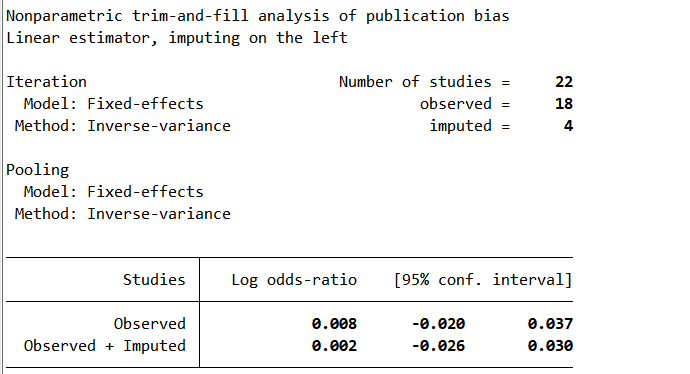


1. summer vs. autumn


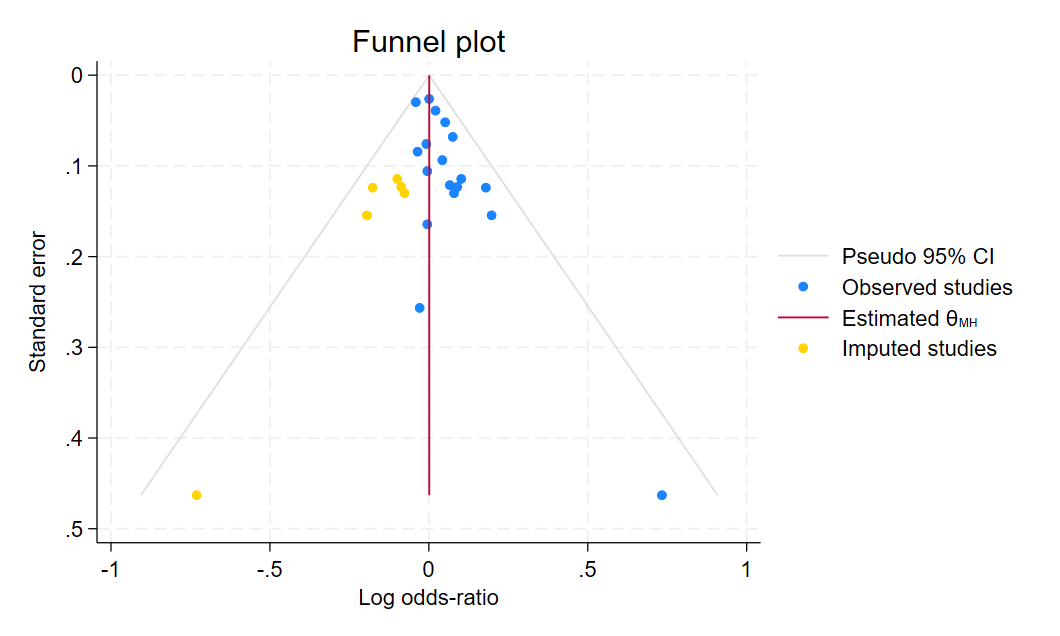


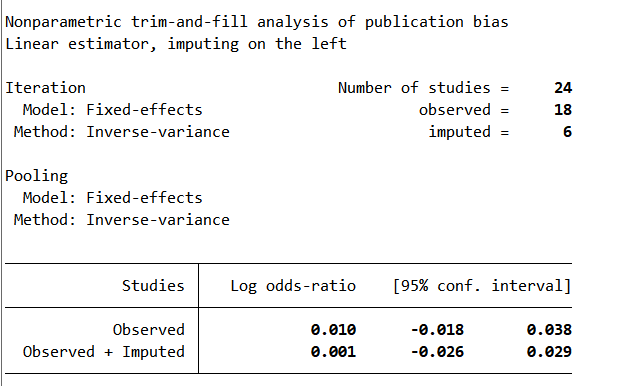


Figure S3. Sensitivity analysis - clinical pregnancy


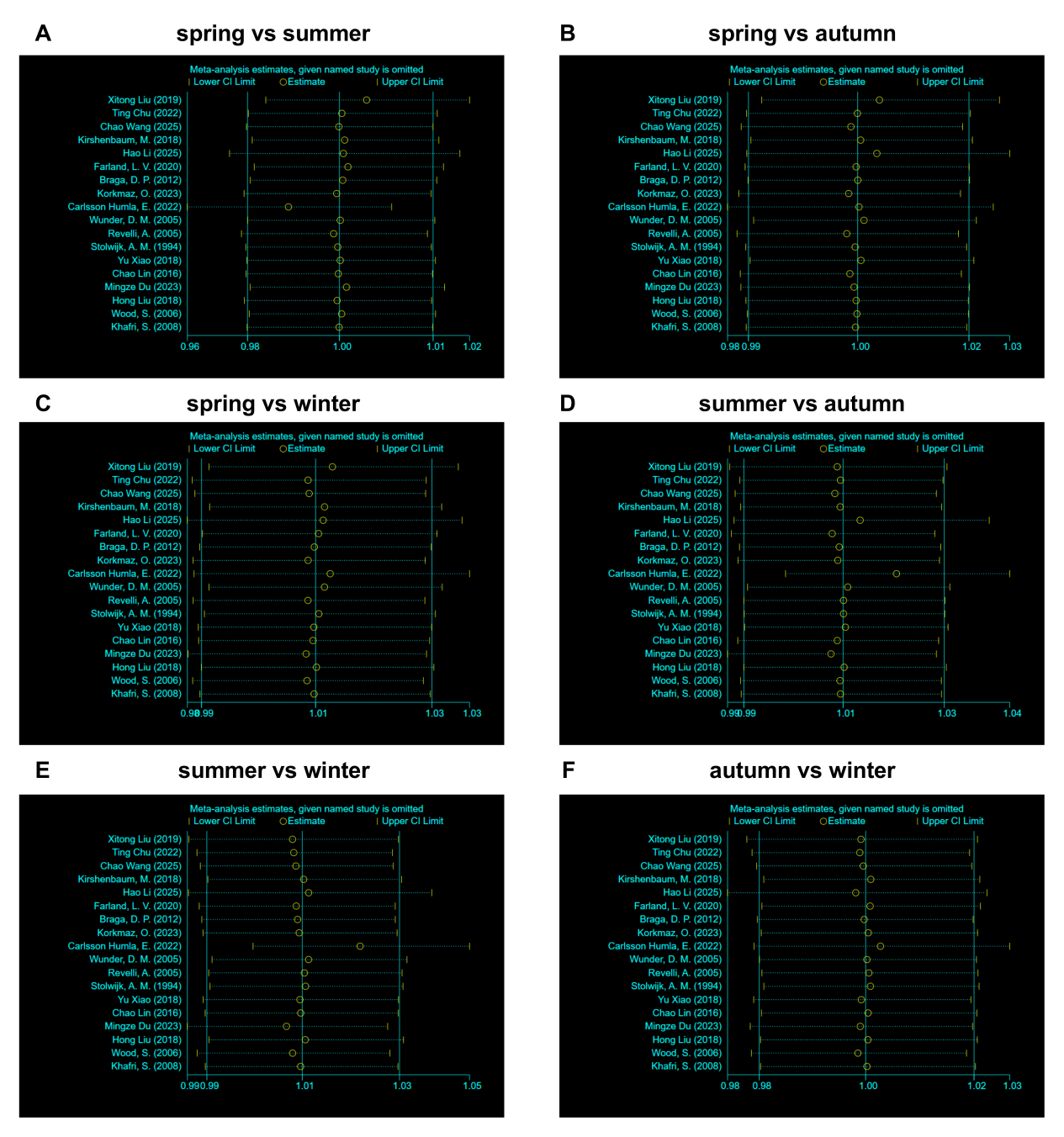


Figure S4. Sensitivity analysis - live birth


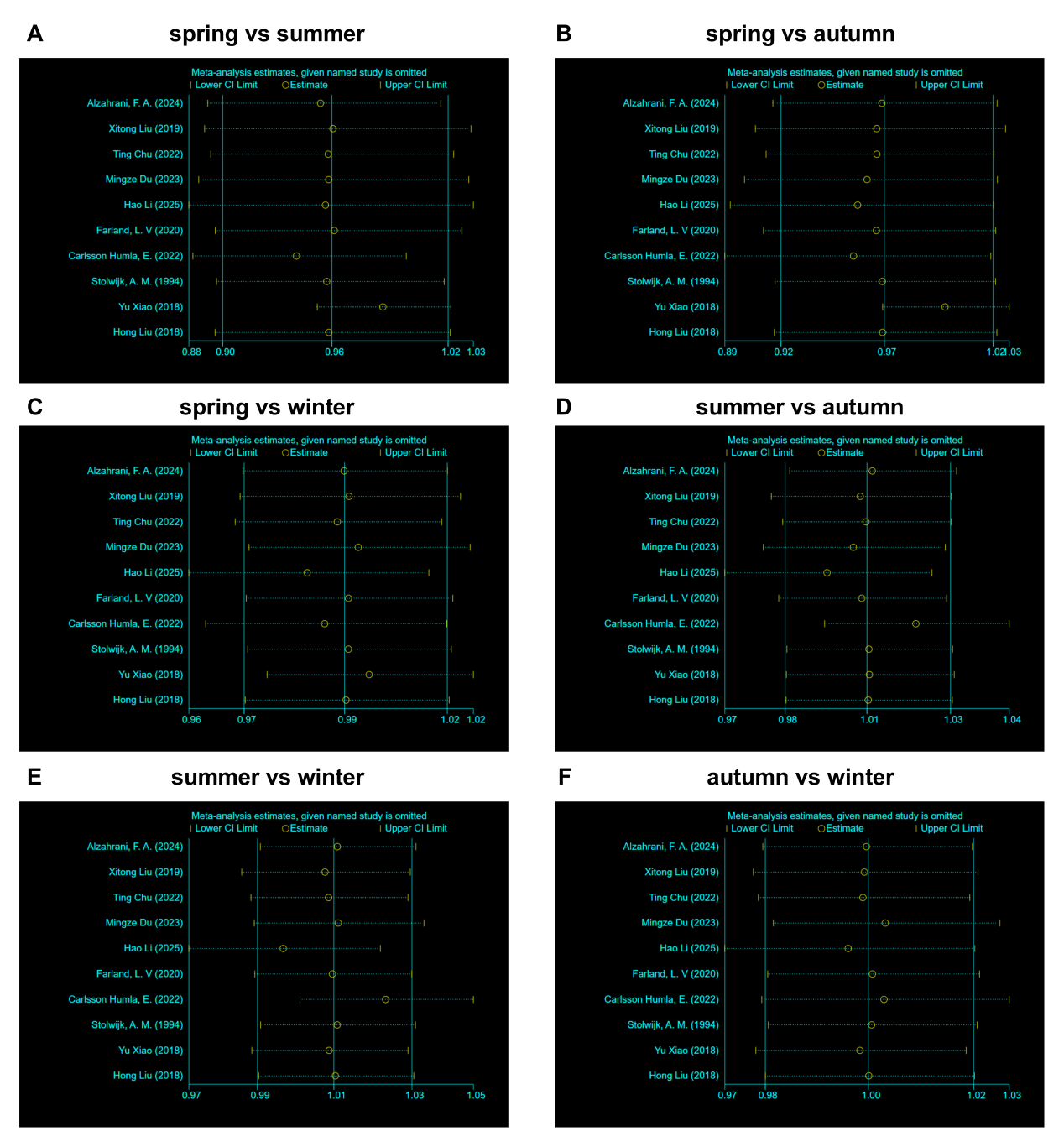

Supplement: Supplementary file 1 [file Data_Sheet_1.doc]
